# Association between exposure to ambient air pollution and occurrence of inflammatory acne in the adult population

**DOI:** 10.1186/s12889-021-11738-0

**Published:** 2021-09-14

**Authors:** Claudia El Haddad, Nour-Estelle Gerbaka, Souheil Hallit, Carmen Tabet

**Affiliations:** 1 Faculty of Medicine and Medical Sciences, Holy Spirit University of Kaslik (USEK), Jounieh, Lebanon; 2 Research Department, Psychiatric Hospital of the Cross, Jal Eddib, Lebanon; 3 Dermatology Department, Notre-Dame des Secours University Hospital, Byblos, Lebanon

**Keywords:** Acne, Pollution, Lebanon, Adults, Association

## Abstract

**Background:**

Acne vulgaris is one of the most prevalent skin diseases responsible for dermatological consultations. Several internal and external factors can affect acne occurrence and severity. Outdoor air pollution is an external factor discussed to trigger inflammation of the skin. The objective of this study was to find a link between the exposure to ambient air pollution and inflammatory acne occurrence in the Lebanese adult population.

**Methods:**

A cross-sectional study was conducted, using an online questionnaire to collect the required data from different Lebanese regions. The survey covered pollution exposure questions as well as queries on several factors known to have a role on acne occurrence.

**Results:**

A total of 372 participants were included in the study, aged 18 to 55 years old. The results of a logistic regression taking the presence/absence of acne as the dependent variable, showed that female gender (aOR = 4.39), younger age (aOR = 1.05), using hydrating cream (aOR = 4.30), working near a power plant vs not (aOR = 3.07), having a severe NO2 exposure compared to none (aOR = 8.24), a higher number of family members with acne or history of acne (aOR = 1.48) were significantly associated with higher odds of having acne, whereas having a dry skin compared to normal (aOR = 0.20) was significantly associated with lower odds of having acne.

**Conclusion:**

The occurrence of inflammatory acne in Lebanese adults was found to be associated with ambient exposure to high levels of NO_2_ and employment near a power plant known to emit CO_2_, CO, SO_2_, NO_2_ and PM. Therefore, our findings can serve as a first step towards implementing awareness on a skin care routine suitable for highly polluted areas.

**Supplementary Information:**

The online version contains supplementary material available at 10.1186/s12889-021-11738-0.

## Background

Acne vulgaris is a chronic inflammatory multifactorial skin disease involving the pilosebaceous unit. It is the 8th most prevalent disease in the world [1]. A study conducted in US Alabama [2], indicates that 73% of people aged 20 years and older stated ever having acne. Epidemiological studies had shown that it is a skin disease known to reach a peak during teenage years but can keep on being a problem in adulthood [3]. In Lebanon, a retrospective study showed that Acne Vulgaris was the most prevalent skin disease among patients between 16 and 33 years of age [4].

The multifactorial pathogenesis of acne consists of increased sebum production, inflammation, altered keratinization and bacterial colonization of the hair follicle by *Cutibacterium Acnes* formerly known as *Propionibacterium acnes*. In spite of symptoms such as pain and stinging, acne alone is considered as a benign non-life-threatening disease with no effect on general physical health. Nevertheless, it can generate serious psychological and social consequences due to visible erythematous pustules, papules, and in severe cases nodulocystic lesions, as well as the residual scaring and hyperpigmentation it leaves. Concomitant anxiety, reduced self-esteem, distorted body image, and signs of depression were significantly found in people with acne [5]. With today’s world highlighting more and more social appearances, it has become a necessity and a challenge for one to always look his or her best.

Previous studies showed relevant association between acne occurrence and gender, some stated it being more prevalent women, other more common in man, and some stated no difference in sexes [6]. As we may well know, sebum production and secretion are stimulated by androgens in an autonomous endocrine gland manner. Thereby, hormonal factors can contribute to the pathogenesis of acne. Paradoxically, estrogens suppress sebum production [7].

In an article by Dreno and Poli [8], higher prevalence of acne was reported in patients with a positive family history of acne. In addition, skin type, which is classified according to an individual’s skin sebum level, is considered to have a role in acne occurrence. In fact, *Cutibacterium acnes* has a predilection for environments with high sebum levels, thus oily and mixed skin are believed to increase the risk of acne presentation [6]. Moreover, nutrition was assumed for many years to cause or exacerbate acne, but recent studies using the food frequency questionnaire revealed the only association to arise from dairy products, hyperglycemic food, carbohydrates, whey protein and chocolate [9]. Overweight, obesity and high BMI level resulting from the latter types of food have been as well found to be associated with acne [9]. Furthermore, in the study by B. Dreno and C. Taïeb about myriad acne exposomes [10], a significant number of individuals with acne were found suffering from stress and lack of sleep. Exposure to screens and tablets daily for a notable amount of time was reported in individuals of both groups, with more individuals in the acne group (91.3%) vs acne-free group (85.2%). Contraceptive use was more common in individuals suffering from acne. A single-blinded randomized clinical trial [11] elicited the importance of facial cleansing twice daily; in point of fact, when individuals abstain from washing their face twice daily, and adapted only a once daily routine, a significant increase in erythema, papules and inflammatory lesions was observed. However, facial medical devices for cleansing were reported being used by 35% of individuals with acne, compared to 16.7% in acne-free group [10]. Heavier creams can worsen congestion of pores in the face leading to inflammation of the sebaceous gland. Hence, the use of hydrating creams not suited for one’s skin type may worsen acne lesions or initiate their occurrence.

Skin is the interface between the body and the outside world. Its primary role is to provide an external protection for our body from any chemical or physical elements, thus it’s considered the barrier with environmental factors. Ambient air pollution is an essential problem worldwide [12]. In 2016, 91% of the world population was living in regions where the WHO air quality guidelines were not met [12]. Outdoor air pollution was estimated to cause 4.2 million premature deaths in the year of 2016, with the highest percentage being uphold by the Asian continent [12]. During the past few years, Lebanon, a Mediterranean country portrayed by its lack of infrastructure and public transportation, witnessed severe pollution events, especially worth mentioning is the waste crisis during the year of 2019. In fact, different studies investigating air quality over the region of the Mediterranean basin emphasized the region as being one of the air pollution hotspots [13]. The main outdoor pollutants, as defined by the United States Environmental Protection Agency (US EPA), extract from gaseous compounds, which are nitrogen dioxide (NO_2_), Sulphur dioxide (SO_2_), carbon monoxide (CO), heavy metals, particulate matter 10 (PM_10_) and particulate matter 2.5 (PM_2.5_) [14]. Additionally, nitrogen oxide compounds interact with volatile organic compounds (VOCs) upon ultraviolet (UV) photoactivation to generate ground-level ozone (O_3_) [14]. Air in Lebanon is characterized by high levels of ozone during summer due to the cloud-free conditions, high solar radiation intensity and the fact that this precise region is at the intersections of polluted air masses from Europe, Asia and Africa [13]. Particulate Matter (PM) is similarly considered a major pollutant of the region resulting from numerous activities related to industrial and transport sectors in urban areas [13].

Several studies draw evidence of a relationship between air pollution and various health outcomes especially cardiovascular [15] and respiratory [16] diseases. As for the skin, previous studies [3, 17] and review articles [18, 19] have provided robust evidence of relevant skin changes following exposure to ambient air pollutants, specifically the occurrence of inflammatory acne [19]. The trigger of an oxidation stress response in human skin was observed as the outcome of air pollutants exposure: relevant antioxidants of the skin such as Vitamin E and Squalene were found to be reduced as well as ATP levels, and oxidized protein levels increased, following exposure to high levels of outdoor pollution [18]. The oxidation will cause keratinocyte hyperproliferation and inflammatory cytokine release, leading ultimately to the onset or worsening of acne [3].

The total quantity of studies performed over the past years discussing correspondences of health conditions with the environment in Lebanon is underwhelming, despite the unfavorable quality of many environmental features especially air. Recent studies showed an association between pollution and chronic diseases (hypertension, cardiac problems, and stroke) [20–22]. Our study discusses the deleterious effects air pollutants induce chronically on the skin. Precisely, it aims to investigate the association between exposure to ambient air pollution and occurrence of inflammatory acne in the adult population.

## Methods

### General study design

A cross-sectional study design was used to help determine if exposure to outdoor air pollution is associated with acne. Between March and August 2020, due to the Covid19 outbreak, a survey was carried out using an anonymous online platform. Data was collected using a snowball sampling method targeting people who lived in regions where Air Quality Monitoring stations (AQMS) are based. The questions were filled by recalling the year 2019, knowing that air pollution faced important changes during the year 2020. People who participated in our study were divided into groups: patients with presence of acne and patients with no acne. Participants were asked to state if their condition was diagnosed by a dermatologist. Participants aged 18 years and older were included. Participants aged less than 18 years, who spent the year 2018 and 2019 abroad, who used medication that caused acne occurrence (lithium, glucocorticoids) were excluded from the study.

### Minimum sample size calculation

The Epi-info software assumed a minimal sample size of 160 participants, based on a percentage of acne in nonsmokers of 70.7% and an OR = 4.14 based on a previous study [10], an alpha error of 5% and a power of 90%.

### Survey development

The questionnaire comprised 70 questions. A pilot study was done on 20 people from different age range prior to finalizing and distributing the survey to ensure the clarity and coherence of the questions. No modifications were added following the pilot study; thus, the answers were included in the study.

The queries were organized into 4 parts. The first part assessed gender, age, socio-demographic questions, and the presence or absence of acne; in addition, participants were asked to name their region of living, as well as their work and university locations if it applies. The second part, the LEEDS scale for acne grading [23], a pictorial validated scale, was to be filled by participants who stated having acne and was used to assess acne severity over face, back and chest. The third part comprised queries about diverse acne risk factors believed to play a role in its incidence, such as skin type, stress, nutrition [9] and use of hydrating creams, facial medical devices and certain medications. These questions were based on a previous article [10]. As for the fourth and final part, it assessed exposure to ambient air pollution by living close (< 100 m) to a busy road or a generator, living or working near (< 1 km away) a power plant, driving and using air conditioning in the car, as well as hours spent per day at home, work, university, in traffic and practicing outdoor activities; to note that power plants in most cities in Lebanon are coal-based thermal power plants [21]. Based on the participant’s home, work and university regions, we stratified exposure as None: residency in non-polluted cities, Mild: limited time spent during the day in a polluted city (less than 4 h per day), Moderate: 2 out of the 3 regions (home, work, university) documented as polluted based on each air pollutants percentages, and Severe: 3 out of 3 regions have high levels of the air pollutant in question.

### Assessment of exposure

**Fig. 1:**
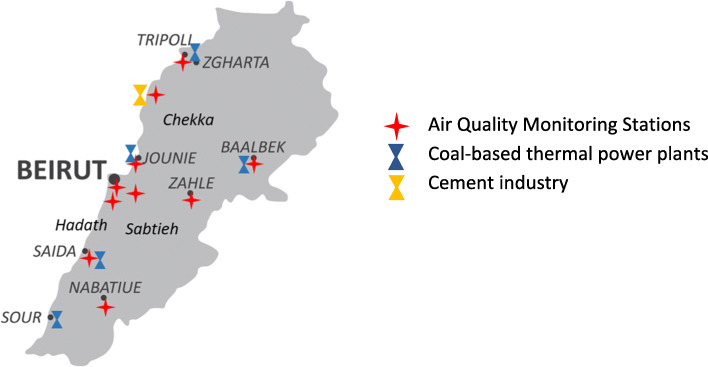
Map of Lebanon illustrating the location of Air Quality Monitoring Stations as well as power plants situated in the cities of interest for this study [26, 27]

Based on the Harvard School of Public Health article on Assessment of Human Exposure to Air Pollution [24], there are three aspects of exposure important to determine related health consequences: Magnitude (the pollutant concentration), Duration (how long the exposure lasts), Frequency (how often the exposure occurs). The remainder two were estimated following the questions on self-reported “hours spent per day” at home, university and work, in the final part of the questionnaire. As regards to the Magnitude, data from the years of 2018 and 2019 were obtained from the national Air Quality Monitoring Network in the Lebanese Ministry of Environment. To further elucidate, there are fifteen Air Quality Monitoring stations (AQMS) using online analyzers to record air concentration of the pollutants considered as the most relevant pollution indicators according to the WHO: CO, NO_2_, O_3_, SO_2_, PM_10_, PM_2.5_ [25]. The stations are installed in specified areas of concern to monitor population exposure to industries, power plants, road traffic in addition to urban sources of pollution (Fig. 1).

Comparison was based on data covering criteria pollutants concentration during June 2018–June 2019 retrieved from the Ministry. These records comprised validated annual averages of 5 regions: Hadath, Beirut, Zahle, Sidon and Baalbeck. The remaining information were raw annual concentrations describing the pollutants in Nabatieh, Tripoli, Zouk, Chekka, and Sabtieh. The regions were chosen based on the data retrieved from the study survey during data collection. The obtained data is listed in Table 1 below.

**Table 1:** Annual mean concentration of each pollutant in specific regions during June 2018–2019

	NO2 (μg/m^3^)	CO (mg/m^3^)	SO2 (μg/m^3^)	O3 (μg/m^3^)	PM10 (μg/m^3^)	PM2.5 (μg/m^3^)
**HADATH**	42.36	0.55	12.92	X	41.16	31.54
**BEIRUT**	48.7*	0.602*	6.23*	25	34.5	25.36
**ZAHLE**	49.2	0.845	4.33	X	40.7	32.7
**SIDON**	37.8	0.491	3	X	44	34.18
**BAALBECK**	18.6	X	X	56.5	31.45	24
**NABATIEH**	11	0.53	4.25	63.4	27.35	16.1
**TRIPOLI**	24	0.73	8.6	63.9	24.43	15.28
**USEK/ZOUK**	46.7	0.557	10.09	43	18.31	11.81
**CHEKKA**	28.9	0.445	13.03	70.97	18.8	11.75
**SABTIEH**	51.2	0.23	10.7	39.63	22.89	13

X = not available; * = non-validated Beirut concentrations.

All pollutants concentrations were stratified based on the World Health Organization guidelines for particulate matter, ozone, nitrogen oxide and sulfur dioxide [28] revised in the year of 2005.

Table 2 illustrates the World Health Organization guidelines updated in 2005 [12] for NO_2_, SO_2_, CO, O_3_, PM_2.5_ and PM_10_ concentrations; as well as the limit values implemented by the Lebanese Ministry of Environment [29]. The thresholds listed are not to be exceeded in order to prevent health damage [12].

**Table 2:** Limit concentration of each air pollutant according to the WHO and to the Lebanese Ministry of Environment

Outdoor air pollutants	WHO guidelines [12]	Lebanese Ministry of Environment averages [29]
NO_2_	40 μg/m^3^ annual mean 200 μg/m^3^ 1-h mean	100 μg/m^3^ annual mean 150 μg/m^3^ 24-h mean 200 μg/m^3^ 1-h mean
SO_2_	20 μg/m^3^ 24-h mean 500 μg/m^3^ 10-min mean	80 μg/m^3^ annual mean 120 μg/m^3^ 24-h mean 350 μg/m^3^ 1-h mean
CO	10 mg/m^3^ 8-h mean 30 mg/m^3^ 1-h mean	10,000 μg/m^3^ 8-h mean 30,000 μg/m^3^ 1-h mean
O_3_	100 μg/m^3^ 8-h mean	100 μg/m^3^ 8-h mean 150 μg/m^3^ 1-h mean
PM_2.5_	10 μg/m^3^ annual mean 25 μg/m^3^ 24-h mean	–
PM_10_	20 μg/m^3^ annual mean 50 μg/m^3^ 24-h mean	80 μg/m^3^ 24-h mean

### Variables

In our survey, we assessed family history, presence of stress or sleep deprivation, use of creams and medical devices by Yes or No questions. Long term exposure to screens was evaluated by stating the number of hours spent per day in front of a screen. As for diet, weekly consumptions of sugar-based products, whey proteins, dairy products and carbs were asked. The same weekly evaluation was applied for face washing. Also, participants were to state their skin type, and to specify if the statement was based on a dermatologist’s diagnosis or not.

### Statistical analysis

The SPSS software v.25 was used for all statistical analysis. The Chi-square and Fisher exact tests were used to compare categorical variables, whereas the Student t test was used to compare two means. Logistic regressions were conducted taking the dermatologist-diagnosed acne (yes/no) and the LEEDS score (≥2 vs < 2) as dependent variables. Variables that showed a *p* < 0.2 in the bivariate analysis were considered as independent variables in the final model (gender, age, use of hydrating cream, passive smoking, working near a power plant, NO_2_ exposure categories, number of family members with acne or history of acne, skin type). Significance was set at *p* < 0.05.

## Results

### Sociodemographic and other characteristics of the participants

A total of 372 participants aging from 18 to 55 years old completed the questionnaire. The mean age of the participants was 29.44 ± 12.71 years, with 67.2% females. In addition, 124 (33.3%) had acne diagnosed by a physician. All details about other sociodemographic and characteristics of the sample are summarized in Table 3.

**Table 3:** Sociodemographic and other characteristics of the participants (*N* = 372)

Variable	N (%)
**Gender**	
Male	122 (32.8%)
Female	250 (67.2%)
**District**	
Beirut	62 (16.7%)
Mount Lebanon	271 (72.8%)
North Lebanon	27 (7.3%)
South Lebanon	9 (2.4%)
Bekaa	3 (0.8%)
**Acne diagnosis by a dermatologist**	
No	248 (66.7%)
Yes	124 (33.3%)
**LEEDS score for the face**	
< 2	291 (78.2%)
≥ 2	81 (21.8%)
**LEEDS score for the back**	
< 2	340 (91.4%)
≥ 2	32 (8.6%)
**LEEDS score for the chest**	
< 2	353 (94.9%)
≥ 2	19 (5.1%)
**Total LEEDS score (face, back and chest)**	
< 2	278 (74.7%)
≥ 2	94 (25.3%)
**Number of times you wash your face per week**	
Never	247 (66.4%)
1–2 times	47 (12.6%)
3–5 times	46 (12.4%)
Daily	32 (8.6%)
**Skin type**	
Normal	88 (23.7%)
Dry	25 (6.7%)
Mixed	139 (37.4%)
vOily	90 (24.2%)
Do not know	30 (8.1%)
**Use of hydrating cream (yes)**	266 (71.5%)
**Use of medical devices (yes)**	31 (8.3%)
**Use of cream powder (yes)**	111 (29.8%)
**Stress/sleep deprivation (yes)**	174 (46.8%)
**Intake of sugar-based products**	
1–2 times per week or less	121 (32.5%)
3–5 times per week	135 (36.3%)
Every day	116 (31.2%)
**Whey proteins intake**	
1–2 times per week or less	343 (92.2%)
3–5 times per week	22 (5.9%)
Every day	7 (1.9%)
**Dairy products intake**	
1–2 times per week or less	64 (17.2%)
3–5 times per week	157 (42.2%)
Every day	151 (40.6%)
**Carbohydrates intake**	
1–2 times per week or less	154 (41.4%)
3–5 times per week	154 (41.4%)
Every day	64 (17.2%)
**Hours in front of screen**	
0–1 h	8 (2.2%)
2–3 h	95 (25.5%)
4–5 h	133 (35.8%)
6 h or more	136 (36.6%)
**Smoking (yes)**	124 (33.3%)
**Passive smoking daily (yes)**	167 (44.9%)
**Living close to a highway/busy road (yes)**	119 (32.0%)
**Living close to an electric generator (yes)**	94 (25.3%)
**Living near a power plant (yes)**	42 (11.3%)
**Working near a power plant (yes)**	24 (6.5%)
**Driving a car (yes)**	328 (88.2%)
**Using the air condition in the car (yes)**	352 (94.6%)
**Hours stuck in traffic per day**	
Not applicable	85 (22.8%)
1–2 h per day	243 (65.3%)
3–4 h per day	40 (10.8%)
5 or more hours per day	4 (1.1%)
**Hours of outdoor activities per day**	
Not applicable	91 (24.5%)
v1–2 h per day	203 (54.6%)
v3–4 h per day	69 (18.5%)
5 or more hours per day	9 (2.4%)
	**Mean ± SD**
**Age (in years)**	29.44 ± 12.71
**Body Mass Index (kg/m**^**2**^**)**	23.32 ± 4.57
**Household crowding index**	0.93 ± 0.43
**Number of family members with acne or history of acne**	0.73 ± 0.76
**Exposure to NO**_**2**_**categories**	
None	35 (9.4%)
Mild	113 (30.4%)
Moderate	180 (48.4%)
Severe	44 (11.8%)
**Exposure to CO categories**	
None	354 (95.2%)
Mild	16 (4.3%)
Moderate	2 (0.5%)
Severe	0 (0%)
**Exposure to SO**_**2**_**categories**	
None	273 (73.4%)
Mild	75 (20.2%)
Moderate	22 (5.9%)
Severe	2 (0.5%)
**Exposure to O**_**3**_**categories**	
None	328 (88.2%)
Mild	43 (11.6%)
Moderate	1 (0.3%)
Severe	0 (0%)
**Exposure to PM**_**10**_**categories**	
None	121 (32.5%)
Mild	129 (34.7%)
Moderate	105 (28.2%)
Severe	17 (4.6%)
**Exposure to PM**_**2.5**_**categories**	
None	4 (1.1%)
Mild	88 (23.7%)
Moderate	213 (57.3%)
Severe	67 (18.0%)

### Bivariate analysis

In a bivariate analysis comparison, higher percentages of females in contrast to males (38.4% vs 23.0%), an oily skin type in contrast to other skin types, the use of a hydrating cream in contrast to no use of hydrating creams (38.3% vs 20.8%), stress in contrast to no stress (38.5% vs 28.8%), suffered from acne. Moreover, a significantly higher mean amount of family members with acne or history of acne (0.97 vs 0.61) was found in participants who present acne lesions (Table 4).

**Table 4:** Bivariate analysis of factors associated with the presence/absence of acne according to the dermatologist’s diagnosis

Variable	Absence of acne (***N*** = 248)	Presence of acne (***N*** = 124)	***p***
**Gender**			**0.003**
Male	94 (77.0%)	28 (23.0%)	
Female	154 (61.6%)	96 (38.4%)	
**Skin type**			**< 0.001**
Normal	77 (87.5%)	11 (12.5%)	
Dry	19 (76.0%)	6 (24.0%)	
Mixed	85 (61.2%)	54 (38.8%)	
Oily	48 (53.3%)	42 (46.7%)	
**Use of hydrating cream**			**0.001**
No	84 (79.2%)	22 (20.8%)	
Yes	164 (61.7%)	102 (38.3%)	
**Number of times you wash your face per week**			0.207
Never	156 (63.2%)	91 (36.8%)	
1–2 times	35 (74.5%)	12 (25.5%)	
3–5 times	35 (76.1%)	11 (23.9%)	
Daily	22 (68.8%)	10 (31.3%)	
**Use of medical devices**			0.596
No	226 (66.3%)	115 (33.7%)	
Yes	22 (71.0%)	9 (29.0%)	
**Use of cream powder**			0.149
No	180 (69.0%)	81 (31.0%)	
Yes	68 (61.3%)	43 (38.7%)	
**Stress/sleep deprivation**			**0.047**
No	141 (71.2%)	57 (28.8%)	
Yes	107 (61.5%)	67 (38.5%)	
**Intake of sugar-based products**			0.354
1–2 times per week or less	76 (62.8%)	45 (37.2%)	
3–5 times per week	96 (71.1%)	39 (28.9%)	
Every day	76 (65.5%)	40 (34.5%)	
**Whey proteins intake**			0.548
1–2 times per week or less	227 (66.2%)	116 (33.8%)	
3–5 times per week	15 (68.2%)	7 (31.8%)	
Every day	6 (85.7%)	1 (14.3%)	
**Dairy products intake**			0.561
1–2 times per week or less	39 (60.9%)	25 (39.1%)	
3–5 times per week	107 (68.2%)	50 (31.8%)	
Every day	102 (67.5%)	49 (32.5%)	
**Carbohydrates intake**			0.258
1–2 times per week or less	98 (63.6%)	56 (36.4%)	
3–5 times per week	110 (71.4%)	44 (28.6%)	
Every day	40 (62.5%)	24 (37.5%)	
**Hours in front of screen**			0.683
0–1 h	5 (62.5%)	3 (37.5%)	
2–3 h	67 (70.5%)	28 (29.5%)	
4–5 h	84 (63.2%)	49 (36.8%)	
6 h or more	92 (67.6%)	44 (32.4%)	
**Smoking**			0.139
No	159 (64.1%)	89 (35.9%)	
Yes	89 (71.8%)	35 (28.2%)	
**Passive smoking daily**			0.161
No	143 (69.8%)	62 (30.2%)	
Yes	105 (62.9%)	62 (37.1%)	
**Living close to a highway/busy road**			0.116
No	162 (64.0%)	91 (36.0%)	
Yes	86 (72.3%)	33 (27.7%)	
**Living close to an electric generator**			0.933
No	185 (66.5%)	93 (33.5%)	
Yes	63 (67.0%)	31 (33.0%)	
**Living near a power plant**			0.728
No	219 (66.4%)	111 (33.6%)	
Yes	29 (69.0%)	13 (31.0%)	
**Working near a power plant**			0.179
No	235 (67.5%)	113 (32.5%)	
Yes	13 (54.2%)	11 (45.8%)	
**Driving a car**			0.650
No	28 (63.6%)	16 (36.4%)	
Yes	220 (67.1%)	108 (32.9%)	
**Using the air condition in the car**			0.745
No	14 (70.0%)	6 (30.0%)	
Yes	234 (66.5%)	118 (33.5%)	
**Hours stuck in traffic per day**			0.266
Not applicable	61 (71.8%)	24 (28.2%)	
1–2 h per day	159 (65.4%)	84 (34.6%)	
3–4 h per day	24 (60.0%)	16 (40.0%)	
5 or more hours per day	4 (100.0%)	0 (0%)	
**Hours of outdoor activities per day**			0.142
Not applicable	62 (68.1%)	29 (31.9%)	
1–2 h per day	140 (69.0%)	63 (31.0%)	
3–4 h per day	43 (62.3%)	26 (37.7%)	
5 or more hours per day	3 (33.3%)	6 (66.7%)	
**Exposure to NO**_**2**_**categories**			0.079
None	26 (74.3%)	9 (25.7%)	
Mild	65 (57.5%)	48 (42.5%)	
Moderate	128 (71.1%)	52 (28.9%)	
Severe	29 (65.9%)	15 (34.1%)	
**Exposure to CO categories**			0.638
None	235 (66.4%)	119 (33.6%)	
Mild	12 (75.0%)	4 (25.0%)	
Moderate	1 (50.0%)	1 (50.0%)	
**Exposure to SO**_**2**_**categories**			0.735
None	180 (65.9%)	93 (34.1%)	
Mild	53 (70.7%)	22 (29.3%)	
Moderate	14 (63.6%)	8 (36.4%)	
Severe	1 (50.0%)	1 (50.0%)	
**Exposure to O**_**3**_**categories**			0.355
None	220 (67.1%)	108 (32.9%)	
Mild	28 (65.1%)	15 (34.9%)	
Moderate	0 (0%)	1 (100.0%)	
**Exposure to PM**_**10**_**categories**			0.144
None	72 (59.5%)	49 (40.5%)	
Mild	90 (69.8%)	39 (30.2%)	
Moderate	72 (68.6%)	33 (31.4%)	
Severe	14 (82.4%)	3 (17.6%)	
**Exposure to PM**_**2.5**_**categories**			0.182
None	2 (50.0%)	2 (50.0%)	
Mild	51 (58.0%)	37 (42.0%)	
Moderate	150 (70.4%)	63 (29.6%)	
Severe	45 (67.2%)	22 (32.8%)	
**Age (in years)**	31.10 ± 13.89	26.13 ± 9.14	**< 0.001**
**Body Mass Index**	23.99 ± 4.70	21.99 ± 3.99	**< 0.001**
**Household crowding index**	0.94 ± 0.44	0.90 ± 0.44	0.426
**Number of family members with acne or history of acne**	0.61 ± 0.71	0.97 ± 0.82	**< 0.001**
**Times you wash your face per week**			

Numbers in bold indicate significant *p*-values.

### Multivariable analysis

The results of a logistic regression taking the presence/absence of acne as the dependent variable, showed that female gender (aOR = 4.39), younger age (aOR = 1.05), using hydrating cream (aOR = 4.30), working near a power plant vs not (aOR = 3.07), having a severe NO2 exposure compared to none (aOR = 8.24), a higher number of family members with acne or history of acne (aOR = 1.48) were significantly associated with higher odds of having acne, whereas having a dry skin compared to normal (aOR = 0.20) was significantly associated with lower odds of having acne (Table 5).

**Table 5:** Multivariable analysis: Logistic regression taking the presence/absence of acne as the dependent variable

Variable	***p***	aOR	95% CI
Gender (females vs males*)	**0.001**	4.39	1.80–10.68
Age	**< 0.001**	1.05	1.03–1.08
Use of hydrating cream (yes vs no*)	**0.002**	4.30	1.70–10.85
Passive smoking (yes vs no*)	0.069	1.64	0.96–2.79
Working near a power plant (yes vs no*)	**0.039**	3.07	1.06–8.88
NO2 exposure categories (vs no exposure*)	0.105		
Mild	0.149	2.28	0.74–7.02
Moderate	0.436	1.53	0.53–4.43
Severe	**0.022**	8.24	1.36–49.90
Number of family members with acne or history of acne	**0.035**	1.48	1.03–2.13
Skin type	**< 0.001**		
Normal		1	
Dry	**< 0.001**	0.20	0.09–0.44
Mixed	0.103	0.41	0.14–1.20
Oily	0.149	0.61	0.31–1.20

*Reference group; *CI* Confidence Interval; *aOR* Adjusted odds ratio; Nagelkerke R2 = 35.6%; numbers in bold indicate significant *p*-values. Variables entered in the model: gender, age, use of hydrating cream, passive smoking, working near a power plant, NO2 exposure categories, number of family members with acne or history of acne, skin type

The results of a logistic regression taking the presence/absence of acne as the dependent variable, showed that a higher number of family members with acne or history of acne (aOR = 2.60) and a mixed skin compared to normal (aOR = 2.71) were significantly associated with higher odds of having acne, whereas older age (aOR = 0.96) was significantly associated with lower odds of having acne.

## Discussion

Physical appearance plays a major part nowadays in a person’s self-esteem, as first impressions and social image became predominant concerns. Acne has a direct effect on physical appearance, thus more people are seeking medical attention to treat and control their underlying skin disease, with more studies aiming to find external risk factors to insure optimal control. Our study results revealed that exposure to severe NO_2_ levels and employment near a power plant demonstrate a noteworthy association with the presence of acne. Power plants, after the combustion of fossil fuels, emit mainly CO_2_, CO, SO_2_, NO_2_ and PM. Previous findings indicated that an increased ambient N0_2_ exposure in a single pollutant model was related to an increased number of outpatient visits for acne vulgaris [3]. Although the latent study showed relationship as well with increased exposure to NO_2_ PM_10_ and PM_2.5_ in two pollutants models, our study revealed no association with high PM_10_ and PM_2.5_ exposure as a single model but many individuals in the acne group stated working near a power plant.

Furthermore, La Roche-Posay in Beijing, China [3], investigated exposure to the most frequent industry- and traffic-related air pollutants, that is, PM_10_, PM_2.5_, NO_2_, SO_2_, O_3_ and their association with sebum level in addition to the number of inflammatory and noninflammatory acne lesions. Increased sebum secretion and higher number of acne lesions was shown to be related to higher ambient concentrations of PM_2.5_, PM_10_ and NO_2_ [3]. Nevertheless, increased O_3_ concentrations were significantly associated with lower sebum rate and lower numbers of lesions [3].

Concerning the SO_2_ concentrations in both the epidemiological study in Beijing and the study conducted by la Roche-Posay, higher levels were mentioned to be associated with lower sebum rates, less outpatient visits and decreased number of acne lesions. This emphasizes that the correlation between air pollutants and acne vulgaris exists, highlighting that each air pollutant has a different effect on skin barrier function [3].

A study conducted in Mexico compared several biophysical and biochemical parameters of the skin in individuals living in 2 different cities, Mexico City (considered highly polluted) and Cuernavaca (less exposed to pollution) [17]. The sebum excretion rate (SER) on the central zone of the forehead, and on the lateral zone of the forehead was significantly different between Cuernavaca and Mexico City; demonstrating that increased sebum secretion occurred in highly polluted cities. In our study however, we did not evaluate the influence of air pollution on skin events and its effect on sebum production and excretion. A previous study about effects of several acne exposomes revealed that exposure to air pollution by living in proximity to an airport or an industrial site was more frequently observed in the acne group [10].

Concerning age ranges, we know that acne is a skin condition that affects specially teenagers and people in their early twenties. However, acne affects men and women in markedly different ways without ascertaining which sex is more affected [6]. Some studies claim that acne is more prevalent in women, a few stated men as more susceptible, and others found no difference worth mentioning between both sexes in terms of acne occurrence [6]. The present study, which targeted the adult population, revealed that acne is more common in females. Compared to a study conducted on adults 20 years and older, acne reported among women was significantly higher than men after the teenage years, with the difference in all age groups being statistically significant [2].

Our study showed that the presence of a family member with acne or with a history of acne was associated in our study with the presence of acne in participants when presence of acne was assessed by a Dermatologist’s diagnosis, and when dependent variable was evaluated by the Leeds acne scale. The latter results corroborate the ones from a previous study that has also shown family history as being an aggravating factor in acne [30]. This finding is consistent with other studies summarized in the review article on acne epidemiology which discussed a correlation between acne and heritability, with some considering only parents, and others including siblings, first- or second-degree relatives [6]. A study on acne prevalence and associations with lifestyle, conducted through an online survey in multiple European countries, revealed a direct relationship of acne with presence or history in one or both parents [29].

As for age, it is acknowledged that acne occurs mostly in teenage years, due to pubertal hormones [7]. Our results found that a lower mean age was significantly correlated with presence of acne. Concomitant with our results, in a study on acne prevalence in 7 European countries, acne was highest among patients aged between 15 and 17 years old, and decreased with older age [29].

As discussed previously, a higher level of sebum secretion plays a major role in acne pathogenesis [31]. Grossly, it provides a lipid-rich, anaerobic medium in which *P. acnes* can thrive [31]. Consequently, skin type stands as a main factor in acne occurrence. As featured in our results, having an oily skin compared to normal is associated in a bivariate analysis with increased odds of having acne, and dry skin is correlated with lower odds of this occurrence. In a study conducted in South Korea which reviewed available data on facial sebum secretion and its relationship with acne development, the predominant skin type in the acne patients was the normal type, in contrast to the dry type being reported in the control group [31]. Furthermore, an increase in sebum levels in acne patients was found to be related to an increased concentration of sebum insulin-like growth factor-1 (IGF-1) [31]. By comparing the difference in sebum secretion between adolescent acne and post adolescent acne, no significant distinctions in sebum quantity were documented [31].

### Practical implications

Considering the effect of acne on quality of life [32], the correlation found in our study might serve as a first step towards implementing awareness by health care professionals in Lebanon on the relevance of a suitable skin care routine, prompting the use of skin care products to provide a protective barrier and combat oxidants. This would particularly benefit people living in highly polluted regions such as urban areas, in order to minimize the damaging effect of pollution on their skin. Furthermore, it also insists on the importance of patient’s adherence to treatment. Finally, our study highlighted the pressing issue of air pollution in Lebanon, where simple steps such as establishing public transportation to help reduce air pollution can be implemented by both national authorities and population. This study is an addition to the frame of eco-epidemiological studies investigating health effects of air pollution in Lebanon.

### Limitations

As previously mentioned, the questionnaire was distributed using on online platform; thus, this design did not allow to exclude other acne-like conditions such as rosacea and erythema, even though the presence of acne was required to be diagnosed by a dermatologist in order to be included in the results. No validated survey covering all necessary parameters for this study was found after profound research, a newly developed questionnaire incorporating several validated questions was used. Many information in the survey are self-reported, rather than being gathered from a specialist’s clinic, which is subject to information bias. Furthermore, patients in the acne group were diagnosed by a dermatologist rather than self-reported acne, causing a decrease in the number of cases of acne in our study. Moreover, the questionnaire is a recall of the year 2019 possibly leading to recall bias. It is additionally important to note probable decrease in outdoor air pollution during 2020 due to the Covid19 lockdown. Selection bias is likely, due to snowball sampling technique used to recruit participants. Residual confounding bias is also possible since not all factors associated with acne were taken into consideration in this paper. Finally, the study sample was not selected randomly, and targeted specific regions where pollution characteristics were obtainable, and is therefore potentially subject to sampling bias, not guarantying to be fully representative of the general population.

## Conclusion

Evidence of interaction between air pollutants and acne vulgaris are stipulated in several studies. Pollutants trigger inflammatory pathways and stimulate oxidative stress. These pathways induce diverse reactions on the skin. Home and employment location, skin type, lifestyle, and age are factors associated with the occurrence of acne vulgaris. Our results revealed an association between high exposure to outdoor air pollution and incidence of inflammatory acne. In a today’s world where innovations in eco-friendly technology are booming, and mostly due to a drastic increase in population and air pollutants, outdoor air pollution problematic is more pressing than ever. Furthermore, on a national level, simple steps to help reduce air pollution can be implemented by both national authorities and population, particularly traffic-reducing measures by establishing public transportation. Underlining the significance of the latent issue, are novel studies discussing a credible link between the reduction of outdoor air pollutants in 34 countries and the global decline in vehicle transportations due to the Covid19 lockdown during 2020 [33]. More studies are needed to assess the presence of a link between the decline of ambient pollution in the past year and a contraction in acne incidence.

## Supplementary Information

**Additional file 1.**

## Data Availability

All data generated or analyzed during this study are not publicly available to maintain the privacy of the individuals’ identities. The dataset supporting the conclusions is available upon request to the corresponding author.

## Abbreviations

AQMS: Air quality monitoring stations
CO: Carbon monoxide
NO_2_: Nitrogen dioxide
O_3_: Ozone
SO_2_: Sulphur dioxide
PM_10_: Particulate matter 10 μ
PM_2.5_: Particulate matter 2,5 μ
