# Supplementary material for: Association between exposure to ambient air pollution and occurrence of inflammatory acne in the adult population
Source: BMC Public Health. 2021 Sep 14;21:1664. doi: 10.1186/s12889-021-11738-0 (PMC8439009; doi:10.1186/s12889-021-11738-0)
Supplement: Supplementary file 1 — Additional file 1. [file 12889_2021_11738_MOESM1_ESM.docx]

| **Supplementary Table 1. Multivariable analysis: Logistic regression taking the total LEEDS score (≥2 vs <2) as the dependent variable.** | | | |
| --- | --- | --- | --- |
| **Variable** | ***p*** | **aOR** | **95% CI** |
| Age | **0.007** | 0.96 | 0.93-0.99 |
| Working near a power plant (yes vs no*) | 0.084 | 2.73 | 0.88-8.51 |
| Number of family members with acne or history of acne | **<0.001** | 2.60 | 1.74-3.88 |
| Skin type | 0.131 |  |  |
| Normal |  | 1 |  |
| Dry | 0.494 | 1.60 | 0.42-6.15 |
| Mixed | **0.02** | 2.71 | 1.17-6.31 |
| Oily | 0.086 | 2.19 | 0.90-5.35 |

*Reference group; CI=Confidence Interval; aOR=Adjusted odds ratio; Nagelkerke R2=28.6%; numbers in bold indicate significant p-values.
